# Pharmacist-led osteoporosis review: perceptions of current practice, care gaps, and implementation challenges

**DOI:** 10.3399/BJGPO.2025.0093

**Published:** 2026-06-17

**Authors:** Andrew Sturrock, Meaghan Grabrovaz, Laurna Bullock, Emma M Clark, Tracy L Finch, Shona Haining, Toby Helliwell, Robert Horne, Ian Maidment, Daniel Monk, Claire Pryor, Louise Statham, Zoe Paskins, Corinne Turnbull, Janice McKinley

**Affiliations:** 1 Pharmacy Directorate, Public Services Delivery Scotland, Scotland, UK; 2 School of Healthcare and Nursing Sciences, Northumbria University, Newcastle upon Tyne, UK; 3 School of Medicine, Faculty of Medicine and Health Sciences, Keele University, Staffordshire, UK; 4 Bristol Medical School, University of Bristol, Bristol, and North Bristol NHS Trust, UK; 5 Research & Evidence, North East & North Cumbria Integrated Care Board, Newcastle upon Tyne, UK; 6 Centre for Academic Social Care, Public Health, Community and Primary Care Midland Partnership University NHS Foundation Trust, Staffordshire, UK; 7 Research Department of Practice and Policy, UCL School of Pharmacy, UCL, London, UK; 8 College of Life and Health Sciences, Aston University, Birmingham, UK; 9 School of Health & Society, University of Salford, Salford, UK; 10 School of Pharmacy and Pharmaceutical Sciences, University of Sunderland, Sunderland, UK; 11 Haywood Academic Rheumatology Centre, Midland Partnership University NHS Foundation Trust, Staffordshire, UK; 12 PPI contributor, Royal Osteoporosis Society, Bath, UK

**Keywords:** orthopaedics and rheumatology, qualitative research, osteoporosis, general practice

## Abstract

**Background:**

Adherence to oral bisphosphonates for osteoporosis is poor; the challenges and complexity of follow-up reviews in general practice are implicated as a contributory cause. Clinical pharmacists in general practice are an expanding professional group within the UK NHS workforce and could provide person-centred medicines optimisation interventions.

**Aim:**

To explore clinician and patient perceptions towards a pharmacist-led osteoporosis review, including identifying current practice, care gaps, and implementation barriers and facilitators.

**Design & setting:**

Qualitative interview study with patients, clinical pharmacists, GPs, osteoporosis specialists, and service commissioners.

**Method:**

Semi-structured interviews were transcribed verbatim and analysed thematically, informed by a normalisation process theory approach.

**Results:**

In total, 32 participants were interviewed in 22 one-to-one interviews and four small group interviews. Three themes relevant to the design and implementation of a pharmacist-led osteoporosis follow-up review were identified: dissonant views among patients and healthcare professionals about current provision and needs; pharmacists' suitability for, and role in, osteoporosis care and training needs; and contextual issues affecting implementation of a new pharmacist-led osteoporosis review.

**Conclusion:**

Our study found that current practice with respect to following-up patients initiated on oral bisphosphonate treatment in primary care is variable. Although pharmacists were highlighted as well-placed to conduct osteoporosis reviews, varying views about the need for this were highlighted along with a number of contextual barriers, including lack of financial and policy drivers in primary care, workload challenges, varying pharmacist skills and autonomy, and lack of coordination across the health system.

## How this fits in

Previous research has identified a need to support adherence to bisphosphonates via development of an evidence-based, person-centred medicines optimisation review. There is scope for clinical pharmacists in general practice to deliver such reviews, owing to the ongoing expansion of this professional group in the primary care workforce together with reforms in pharmacist training from 2026. This study identified current practice and beliefs of GPs and pharmacists in primary care, osteoporosis specialists in secondary care, and patients with a recent history of oral bisphosphonate use, related to the design and implementation of a pharmacist-led osteoporosis follow-up review.

## Introduction

Estimates show that 3 775 000 (5.2%) of the UK population is living with osteoporosis, and fragility fractures will increase from 527 000 in 2019 to 665 000 in 2034.^1^ Osteoporosis is the fourth most burdensome chronic disease in the UK with fracture-related costs estimated to increase to £5.9 billion by 2030.^2^

National Institute for Health and Care Excellence (NICE) identified prescribing oral bisphosphonates as clinically and cost-effective first-line pharmacotherapy.^3^ However, adherence is poor with estimates of 1 year persistence rates between 16% and 60%.^4^ NICE also recommend follow-up 12–16 weeks after starting bone strengthening medicines to check tolerance and at 12 months to check adherence;^5^ however, people with lived experience of osteoporosis report follow-up as absent.^6^ Evidence suggests that eliciting and addressing patient concerns in follow-up reviews improves medicines adherence.^7^ Furthermore, the absence of follow-up in practice has been identified by patients as having a significant effect on their decision to discontinue treatment and has therefore been highlighted as a priority area for osteoporosis research.^8^

For a medication to work it must be taken, and supporting adherence to bone strengthening medications, including patients’ readiness, is a key aspect of any medication review.^9,10^ Poor adherence is influenced by combinations of internal and external factors, specific to each patient, which can change over time and vary with different treatments.^9,11^ These factors include fear of side effects, beliefs around necessity of medication or effectiveness, and the treatment burden.^12^

Clinical pharmacists working in primary care already provide support to patients managing long-term conditions and polypharmacy,^13,14^ with patients perceiving these to be acceptable.^15^ New ‘Standards for the Initial Education and Training of Pharmacists’ by the General Pharmaceutical Council will see all pharmacists qualify as independent prescribers on registration from 2026.^16,17^ This will allow pharmacists to take a greater role in clinical assessment and diagnosis, the initiation, optimisation, and escalation of treatment, and supporting patients’ information needs.

This study aimed to explore clinician, patient, and commissioner perceptions towards a pharmacist-led osteoporosis medication review, including identifying current practice, information needs, and implementation barriers and facilitators. Resources have been developed as part of the Improving uptake of Fracture Prevention drug Treatments (iFraP) study and are being trialled,^18^ including a decision support tool (DST) to support clinicians and patients to make informed decisions about bone strengthening medicines and to improve adherence in a secondary care settings. This study builds on this research, to inform adaptions of iFraP resources for use in a primary care setting and the need for and structure of a new pharmacist-led review.

## Method

A qualitative semi-structured interview and small group interview study was conducted to explore clinician and patient perceptions towards a pharmacist-led osteoporosis review, including identifying current practice, care gaps, and implementation barriers and facilitators. One-to-one and small group interviews were undertaken with patients and professionals involved in care. The full protocol is previously published.^9^ This article reports results from the first phase aimed to inform follow-up, review development, and identify issues associated with implementing the review. Normalisation process theory (NPT), a theory for understanding the implementation, embedding, and integration of practices, informed topic guide development, data analysis, and interpretation.^19^ Topic guides provided structure to support initial questions; however, flexibility within the qualitative interviews allowed for the emergence of new themes to be explored during the interview and in subsequent data collection with other participants.

Patient and Public Involvement and Engagement (PPIE) members joined the project team to provide advice and feedback on project aims, design, and data analysis and interpretation (see Supplementary data S1 and Table S1). Members were reimbursed for their time involved in the study.

### Participant recruitment

The following five participant groups were recruited: pharmacists working in primary care; GPs; osteoporosis specialists; patients with a history of oral bisphosphonate use; and service commissioners. Participant recruitment was facilitated via a local National Institute for Health and Care Research (NIHR) Regional Research Delivery Network (RRDN), professional networks of the study team and stakeholders, and a regional Royal Osteoporosis Society group. Clinicians were offered reimbursement for time to participate in interviews at NIHR approved rates and patients were offered £20 shopping vouchers in recognition of the time they contributed.

### Data collection

Data were collected from a total of 32 participants (Table 1); 22 one-to-one semi-structured interviews and 10 small group interviews, based on participant choice and scheduling around participants’ clinical commitments. Small group interviews provided an interactional dynamic between participants, which enriched the data.

**Table 1.** Summary of participant categories and numbers

Participant category	Total number of participants	Small group interview (no. of participants)	One-to-one interview	Additional information
**Pharmacists**	12	1 with 3 participants 1 with 2 participants	7	All working in general practice some or all of the time
**Patients**	8	1 with 3 participants	5	
**GPs**	4	-	4	
**Osteoporosis specialists working in secondary care**	7	1 with 2 participants	5	5 medics; 1 specialist nurse; 1 specialist pharmacist
**Service commissioners**	1	-	1	Service commissioner recruitment was low, likely owing to reorganisation of commissioning organisations’ structures at the time of recruitment. GP and service commissioner data combined to maintain confidentiality
**Total**	**32**	**4 with 10 participants**	**22**	

Patients were asked to share their age, sex, and ethnic group on a voluntary basis with the interviewer. Eight of the patients who participated in this study identified as White British, female, and aged 60–86 years.

Participants were offered a choice of online or face-to-face interviews, and group or one-to-one interviews. Two patient interviews were face-to-face: one in the patient’s home and one in a private room at the patient’s GP surgery. All other interviews were conducted online. Informed consent was obtained from all participants. Data collection took place between October 2023 and August 2024.

One researcher (MG) undertook all data collection, using topic guides (Supplementary data S2) tailored to each participant category and developed by the research team, informed by NPT,^18^ rapid realist review,^6^ the iFraP study,^18^ the PPIE group, and other stakeholders. Topic guides explored current practice, views about a pharmacy review including components identified in the pre-project evidence synthesis,^9^ perceived barriers and facilitators, and training needs for pharmacists

The interviewer also demonstrated a mock-up of an iFraP DST (Supplementary data 3 iFraP tool 1), a review to support decision making about bone strengthening medicines, and asked participants for thoughts and reactions to aspects and applications of the tool.

### Analysis

All interviews except one (recorder failure) were audio-recorded, transcribed verbatim, and anonymised. Data storage, management, and analysis was facilitated by NVivo (verision 12 Pro). Field notes were taken during all interviews and used as a data source for the one interview that was not recorded. Data were inductively analysed by MG using reflexive thematic analysis.^20^ Analysis and interpretations were discussed and refined with the other members of the qualitative research team — AS (principal investigator; PI), MG (senior research assistant), and LB, TF, ZP (co-authors) — in regular team meetings and discussions. In these meetings, inductively developed categories were discussed in relation to NPT constructs to further develop analytical understanding of the work of implementation. Feedback on findings from the PPIE group assisted interpretation of findings from the patient perspective.

## Results

### Dissonant views among patients and healthcare professionals about current provision and needs

Table S2 in Supplementary data 4 provides additional illustrative quotes to demonstrate the different views within and between different participant categories.

Current practice with respect to following-up patients on oral bisphosphonates in primary care is highly variable:

*‘Follow-ups are not particularly well done if I’m completely honest.’* (GP-01)

Not all clinicians believe follow-up is needed, nor that it will improve adherence. Pharmacists in primary care identified that there was no standardised approach to monitoring adherence:

*‘How do you know whether patients are adherent?*’ (Interviewer)

*‘It’s just ad hoc.’* (Pharmacist-10)

Not all clinicians believe there are adherence issues with their patients, and only some GPs and pharmacists use more probing questions to elicit patient concerns or identify potential adherence issues, with some expressing perceptions in interview that it was an area where they lacked knowledge:

*‘I would be really interested actually to have a look and see how many people just take it for a couple of months and then stop. I am not aware of that.’* (Pharmacist-14)

Some pharmacists assume that adherence issues will be picked up at annual review (or ad hoc), despite understanding that prescription ordering history does not necessarily reflect true adherence:

*‘I sort of wait until the next year and see whether, you know, see what their ordering has been like, but that doesn’t always comply with whether or not they’re actually taking it.’* (Pharmacist-04)

Many clinicians believe that ‘*good*’ initiation counselling, safety-netting, and annual reviews are sufficient.

In contrast to clinician beliefs and experiences, not all patients reported experiencing ‘*good*’ initiation counselling or follow-up, and some described unmet information needs. Patients described feeling reluctant to get in touch to address these needs; clinicians confirmed they rarely do so:

*‘... you don't know for sure if you're doing the right thing by contacting them. And when you try to ring, because it’s no appointments … what am I going to do … And yes, they do ring you back but its two or three days later. And by that time, you’ve thought, to hell with this medication, I’m not taking it.’* (Patient-01)

Despite clinicians’ beliefs that adherence issues will be picked up at annual reviews, not all patients receive annual reviews:

*‘No, in an ideal world, every patient should get a medication review yearly. But it doesn’t happen and it’s not part of a QOF register, where the system will flag up that they need a certain blood test … it’s not part of … embedded in the system.’* (Pharmacist-10)

Patients described how decision making about treatment initiation and adherence changes over time, together with their information and support needs. For example, patients described how feeling shocked and anxious after diagnosis meant they focused on rare serious side effects (increasing their anxiety about treatment); all they could see were risks:

*‘... if someone had contacted me, say, two months down the line to just sort of say, have you been tolerating it all right … that might have just been nice to have a bit of follow-up within a certain timeframe of starting it, just to reassure you that it’s actually all right to continue with it, and that there’s nothing to be worried about, the side effects. Because when you read the leaflet that comes with this, there’s quite a ...* [list of side-effects].’ (Patient-08)

Lack of follow-up in primary care leaves many patients with little support for up to 5 years (or until the next dual-energy X-ray absorptiometry [DXA] scan), unless patients actively seek help.

Current practice regarding initiation and follow-up does not always meet patients’ information needs and may not support shared decision making and patient choice, where dissonant views were also reported. Patient choice is conceptualised by clinicians as whether to take or not take the medication, because there is only one first-line treatment. Patients conceptualise patient choice as deciding whether to start and keep taking treatment based on informed choice, as well as understanding which treatments are suitable for them and that there are alternatives if needed:

*‘If I was told there was a choice of medicines … there would be that opportunity to discuss them … Or could make a decision on possibly what would be the best option for me.’* (Patient-05)

### Pharmacists’ suitability for, and role in osteoporosis care and training needs

Table S4 in Supplementary data 4 provides additional illustrative quotes to demonstrate the different views within and between different participant categories.

#### Suitability of pharmacists to undertake osteoporosis review

Clinicians and patients believe that pharmacists could lead an osteoporosis review because of their knowledge of medications, and greater accessibility and time availability (subject to appropriate training):

*‘... the GPs don't have time. I think a pharmacist, you need the knowledge of your background medications and things because it’s older people who are usually on all kinds of stuff. And I think it would feel better that way, because they’re asking you to take a medication, so you need an expert in medication, don’t you?*’ (Patient-01)

However, other patients report that like GPs, pharmacists lack time in addition to a lack of person-centred approach:

*‘I felt that the pharmacist was doing this because they'd been told to. They were rushing, and they didn't have anything to offer me.’* (Patient-09)

The role of pharmacists and what work they do is variable, depending on who they are employed by and their qualifications, background, and experience (which can vary considerably); see Supplementary data 5.

#### Training needs

Pharmacists’ skills are varied because they have varied backgrounds (for example, primary care, community, or hospital), qualifications, and skills. Pharmacists may only get involved in osteoporosis management once a decision to initiate treatment has been made by a GP or in secondary care. Thus, clinical training about osteoporosis treatment options, use of the FRAX**^®^** tool (fracture risk assessment tool) and bone density scan (DXA) scores were identified by some pharmacists as needed to deliver an osteoporosis review, in addition to ‘softer’ skills such as delivering person-centred care:

*‘... more* [training] *about the second-line options potentially, so that, you know, if the patient has more questions or wants to have that choice, then we can talk about it a bit more, and also, I suppose, more about the actual condition and what that sort of means for patients lifelong, and the impact that it can have on them.’* (Pharmacist-13)

*‘... it would be good to have kind of training about like, you know, DXA scans, the kind of T scores. FRAX score you mentioned there, like whether we could use that as like a kind of like a QRISK score almost in terms of how we explain that to patients. I think we’re very good at explaining Q- QRISK and what that actually means, and perhaps FRAX could become kind of that in terms of a patient aid.’* (Pharmacist-02)

*‘I think you need a lot of soft skills to pick up on non-verbal clues and pick up on patients’ acceptance of what you’re saying, and- and understanding of what you’re saying. They’re very good at nodding their head and saying, yes, yes, yes, and they don’t really sure what you mean.’* (Pharmacist-05)

### Contextual issues affecting implementation of a new pharmacist-led osteoporosis review

See Supplementary data S4 for illustrative quotes in Table S5.

#### Prioritisation and incentivisation

National financial incentives in the Quality and Outcomes Framework (QOF) or Investment and Impact Fund (IIF) or localised incentives are described as key in determining prioritisation of pharmacists’ workload in primary care. GPs and pharmacists report that the reduction of nationalfinancial incentives in QOF (in England) is a disincentive for general practice to prioritise follow-up for this patient group:

*‘... osteoporosis used to be in the QOF register … so there was a payment involved … we get a payment for the QOF register, but not for doing a review. There’s no financial reward or anything. You worry that the quality of care those patients get then deteriorates, because they’ve been put on a treatment, but basically never followed up.’* (Pharmacist-01)

Regional organisations, such as integrated care boards (ICBs) and primary care networks ( ownPCNs),^21^ were also reported to influence whether specific streams of work are prioritised, through their strategic goals. ICBs use regional and national monitoring and benchmarking data to prioritise strategic priorities. The autonomy and work that pharmacists do in primary care varies depending on whether they are directly employed by primary care practices or PCNs, and what knowledge, skills, and experience they have (see Supplement 5).

The perception of regional and local prevalence of osteoporosis also influences GP and pharmacist motivation to adopt new ways of working:

*‘As a practice, we’ve not looked at* [follow-up being done by pharmacists] *because it’s not a big workload for us, but if they had the training then yeah, they could certainly do it.’* (GP-02)

#### Practice-level factors

Pharmacists described how work tasks, autonomy, and internal collaboration could differ between different practices depending on different employment models, role variability based on GP priorities and/or directions and availability of other roles in the team; for example, pharmacy technicians:

*‘In the less autonomous practice … mostly the pharmacists that do it but that’s because they’re sent individually by the GPs, patient by patient, to the pharmacy team to do. Whereas in the more autonomous practice we get the letters, we get the results, we do the booking, we call them without an appointment to try and catch them.’* (Pharmacist-12)

Pressure on rooms in general practice can preclude face-to-face appointments with patients, together with pressure from GPs to avoid face-to-face appointments for conditions they consider can be handled by telephone. Pharmacists identified the need for longer appointments for this activity, but this was not always possible:

*‘Length of time … in general practice …* [iFraP style follow-up review] *will need a minimum thirty-minute appointment, and currently I’m struggling to get a twenty-minute appointment. I can fight my way, because of my experience, and background, and position, but … colleagues in general practice, get two minutes, three minutes, in a consultation, if it’s part of an SMR* [structured medication review] *to address this, along with the other fifteen other meds. Not many people get a twenty-minute osteo review. Some people are expected to do it in ten.’* (Pharmacist-01)

At a local level, ways of working that reduce time burdens on GPs can also be an important influence on whether GPs and practices are willing to adopt new ways of working.

#### Coordination of care across primary, secondary, and community care

Gaps between secondary and primary care can mean some patients do not continue treatment after discharge from secondary care. For example, patients may not understand medication treatment needs to be continued, or fragility fractures and/or discharge letters may not be notified or sent to primary care. Thus, patients may not be identified as at risk of osteoporosis and needing treatment or needing medicine reviews.

A lack of joined-up service between healthcare provider services can also affect treatment initiation as patients struggle to access dental check-ups owing to the current shortage of NHS dentists. Clinicians in both primary and secondary care described the need for guidance around initiating treatment in the absence of dental check-ups to avoid delaying treatment initiation:

‘*So, getting them to see a dentist. We just don’t have any NHS dentists. None of our patients have a dentist, it’s a complete nightmare. But in theory, they see an NHS dentist and get a dental check before they start, but seriously they don’t because they just don’t have a dentist, so what do you do?*’ (GP-01)

## Discussion

### Summary

This study found that current practice with respect to following-up patients initiated on oral bisphosphonate treatment in primary care is variable. Although pharmacists were highlighted as well-placed to conduct osteoporosis reviews, varying views about the need for this were highlighted along with a number of contextual barriers, including lack of financial and policy drivers in primary care, workload challenges, varying pharmacist skills and autonomy, and lack of coordination across the health system.

Patients reported that they would value a follow-up consultation because their experiences of initiation are not currently meeting their needs and, importantly, decision making about treatment initiation and their decision-making, information, and support needs change over time. Current practice fails to recognise the dynamic nature of patients’ needs.

Clinicians and patients believe that pharmacists are potentially well-placed to lead an osteoporosis review. However, variability in pharmacists’ knowledge, skills, and experience require additional training about osteoporosis, including explaining risk. Time pressures and the need to adopt a person-centred care approach may also be issues.

The role of pharmacists and what work they do is variable and depends on who they are employed by and their qualifications, background, and experience. Implementation of an osteoporosis review would need to consider financial and policy drivers (including workloads) affecting the strategic priorities of employers of pharmacists in primary care in addition to other contextual barriers.

### Strengths and limitations

The qualitative design of this study facilitated simultaneous in-depth exploration of the views and experiences of the multiple groups with an interest and role in the design and implementation of a follow-up review. Involving the stakeholder group and PPIE representatives in discussion about analysis and emerging results enhanced the trustworthiness and credibility of the interpretation and analysis.^22,23^

We were only able to recruit patients from one geographical region of the UK, with limited diversity in some patient characteristics. Notably, we successfully recruited clinicians and patients from GP practices in a socioeconomically deprived area. These limitations to recruitment may limit the transferability of our findings and future research should focus on underserved groups, including people with lower health literacy levels, learning disabilities, or dementia.

This research will feed into adaptations of iFraP resources, which will be tested with healthcare professionals.

### Comparison with existing literature

A recent review of adherence research in clinical settings identified a need for further research in this area and highlighted four key themes — causes, consequences, mitigation, and methodology — around adherence measurement.^24^ This study contributes to the body of adherence mitigation research through developing a targeted (osteoporosis) personalised adherence intervention, based on a partnership approach between patients and one group of professional healthcare providers.^25^

Although previous research has shown mixed evidence about the effectiveness of pharmacist-led medication reviews in general (for example, Chambers *et al*, Craske *et al*, Jokanovic *et al*, and Wormall and Abbot),^26–29^ pharmacist-based medication counselling has been shown to have potential to improve osteoporosis medication adherence.^30–33^

The clinical pharmacist workforce has been expanding since 2015 in English general practice to alleviate workforce and workload pressures (through elements of role substitution); to increase the quality of prescribing and deprescribing; and to enhance the quality of medication reviews and optimise medication management.^14,34^ This increased workforce availability, as well as expertise in medicines optimisation and greater consultation time availability of practice pharmacists, have been identified as key facilitators for pharmacist-led reviews for other conditions or issues, such as opioid reviews.^35^ However, funding and employment models have been shown to impact on practice pharmacist role negotiation and fulfilment.^13,36^

### Implications for research and practice

These findings provide evidence that an osteoporosis review based on patient-centred, shared decision-making principles to support adherence would meet patients’ currently unmet needs and that clinical pharmacists in general practice could deliver the review (with appropriate training, support and resources, such as a DST). Clinical pharmacists in general practices are usually funded by a PCN or ICB and so there needs to be buy-in and adoption at these higher strategic levels, and clinical pharmacists need to be considered in the design of any osteoporosis review and implementation strategy. Furthermore, evidence is required that any review, including delivered by pharmacists, or others, is clinically effective and improves adherence rates to influence policy and prioritisation. This additional evidence is likely to include health economics data, balancing time and costs (including opportunity costs) to deliver a follow-up review against benefits in terms of avoiding hospital admissions, improved adherence rates, and long-term reduced rates of further fragility fractures.

There is also a need to address health equity issues including developing information resources to explain complex aspects about osteoporosis and treatment, such as risk in relation to side effects and benefits, in different ways appropriate for different groups of patients.

At a patient–practitioner level, these finding have highlighted further training and decision support needs that have informed adaptions to iFraP resources, which will be further tested in future research. Although some practitioners raised the need for ‘counselling checklists’*,* our PPIE representatives were not keen on checklist-based consultations, preferring a more individualised and person-centred approach.
